# Racial/Ethnic Differences in Sexual Network Mixing: A Log-Linear Analysis of HIV Status by Partnership and Sexual Behavior Among Most at-Risk MSM

**DOI:** 10.1007/s10461-014-0842-8

**Published:** 2014-08-01

**Authors:** Kayo Fujimoto, Mark L. Williams

**Affiliations:** 1 Division of Health Promotion & Behavioral Sciences, School of Public Health, The University of Texas Health Science Center at Houston, 7000 Fannin Street, UCT 2514, Houston, TX 77030-5401, USA; 2 Department of Health Policy and Management, College of Public Health & Sciences, Florida International University, 11200 SW 8th Street, Miami, FL 33199-2516 USA

**Keywords:** Social network mixing, Male sex workers, MSM, HIV racial/ethnic disparity, Log-linear model, Risky sexual behavior, Mezcla de redes sexuales, Trabajadores sexuales, HSH, Disparidad racial/étnica, De VIH, Modelo log-lineal, Conducta sexual de riesgo

## Abstract

Mixing patterns within sexual networks have been shown to have an effect on HIV transmission, both within and across groups. This study examined sexual mixing patterns involving HIV-unknown status and risky sexual behavior conditioned on assortative/dissortative mixing by race/ethnicity. The sample used for this study consisted of drug-using male sex workers and their male sex partners. A log-linear analysis of 257 most at-risk MSM and 3,072 sex partners was conducted. The analysis found two significant patterns. HIV-positive most at-risk Black MSM had a strong tendency to have HIV-unknown Black partners (relative risk, *RR* = 2.91, *p* < 0.001) and to engage in risky sexual behavior (*RR* = 2.22, *p* < 0.001). White most at-risk MSM with unknown HIV status also had a tendency to engage in risky sexual behavior with Whites (*RR* = 1.72, *p* < 0.001). The results suggest that interventions that target the most at-risk MSM and their sex partners should account for specific sexual network mixing patterns by HIV status.

## Resumen

Patrones de mezcla en las redes sexuales han mostrado tener un efecto en la transmisión del VIH tanto dentro y a través de grupos. Esta investigación examinó patrones de mezcla sexual relacionados con estatus VIH-desconocido y conducta sexual de riesgo condicionados en mezcla asortativa/disortativa por raza/etnicidad. La muestra empleada para esta investigación consistió de trabajadores sexuales masculinos que consumen drogas y sus parejas masculinas. Un análisis log-lineal se realizó de 257 HSH de más alto riesgo y 3,072 parejas sexuales. El análisis encontró dos patrones significativos. HSH afroamericanos de estatus VIH-positivo de más alto riesgo tenían una fuerte tendencia a tener parejas afroamericanas de estatus VIH-desconocido (riesgo relativo, RR = 2.91, p < 0.001) y de tener conducta sexual de riesgo (RR = 2.22, p < 0.001). HSH blancos de más alto riesgo de estatus VIH-desconocido también tenían una tendencia de tener conducta sexual de riesgo con blancos (RR = 1.72, p < 0.001). Los resultados sugieren que intervenciones dirigidas a HSH de más alto riesgo y sus parejas sexuales deben tomar en cuenta específicos patrones de mezcla en las redes sexuales por estatus de VIH.

## Introduction

In 2010 men who have sex with men (MSM) accounted for 63 % of new human immunodeficiency virus (HIV) infections in the United States and 78 % of new infections among men [1]. Among MSM HIV infection varies by race/ethnicity. Rates of new infections in Blacks/African Americans and Hispanics/Latinos is three times higher than in Whites [2]. Such racial/ethnic disparities cannot be wholly attributed to differences in individual risk behaviors across racial/ethnic groups [3, 4], as few significant differences in sexual behaviors have been found between White and Black MSM. Unprotected anal intercourse (UAI), engaging in commercial sex work, having sex with known HIV-positive partners, or testing for HIV are virtually the same across racial/ethnic groups [4]. Disparities in infection rates across MSM racial/ethnic groups may be attributable to the higher prevalence of HIV and other sexually transmitted infections (STIs) among minority groups. For example, Blacks may be at higher risk for HIV infection than MSM of other racial/ethnic groups [5] simply because HIV infection is higher among Blacks. However, this explanation begs the question of why rates are higher in some racial/ethnic groups than others. A social network perspective may be helpful in answering this question by changing the focus of inquiry from behaviors to the relational characteristics of sexual and drug-injecting networks [6, 7].

Sexual networks among Blacks are characterized by dissortative mixing by network position [7]. Dissortative mixing in this case refers to individuals’ with few partners choosing partners with many partners. Such network mixing patterns facilitate transmission of infectious disease from a network’s high-risk core to the network’s lower risk periphery. Furthermore, differences by racial/ethnicity group in sexual partner selection may partly explain the disproportionate number of HIV infections among Black MSM [8]. Blacks are more likely to select partners from their own racial group than from other races/ethnicities [7, 9, 10], thereby increasing the chances of selecting an HIV-positive partner. Partner selection based on HIV status, or serosorting, may also influence HIV transmission [11, 12]. Studies that examine partnering by HIV status have produced mixed results. HIV-positive MSM are more than twice as likely as HIV-negative MSM to have HIV-positive partners [13]. The prevalence of UAI is higher among HIV-positive partners than among partners who are status unknown and/or HIV-negative [14]. Both behavioral patterns strongly suggest assortative mixing. However, HIV-positive MSM also report UAI with HIV-negative or HIV status unknown partners [15], suggestive of dissortative mixing by serostatus. The reason for these contradictory findings is unclear. One possible explanation may be racial/ethnic differences in the partner selection by HIV status.

The effectiveness of serosorting hinges on one’s knowledge of one’s own and one’s partner’s HIV status. At least one study found that serosorting may not to be protective among Black MSM compared to White and Hispanic MSM [16]. This may be because Blacks tend to delay testing [17] and, consequently, are less likely to know their HIV status [18, 19] than are other racial/ethnic groups [20]. Further, MSM who are HIV-positive but unaware of their status may believe that sex with men of their own race/ethnicity reduces the risk of HIV-transmission [21]. HIV-negative Black MSM are more likely to have UAI with HIV status-unknown partners than with their White counterparts [8].

In summary, previous studies indicate that serosorting is less common among Black MSM than MSM of other racial/ethnic groups. Black MSM also are less likely to know their status (unknown ego’s status) and are more likely to have sexual partners (alters) with unknown status. How the unknown status of individuals (egos) and/or their sex partners (alters) relates to selection of sexual partners and risky sexual behavior has rarely been examined. Few studies have investigated patterns of sexual network mixing by HIV status among MSM who trade sex for money and use drugs. Among MSM, male sex workers engage in behaviors that significantly increase the risk of HIV infection, such as having drug-injecting sexual partners, engaging in unprotected oral sex, engaging in unprotected anal intercourse, having multiple sex partners, and using drugs [22, 23]. Dissortative mixing by trading sex for money was reported to occur in almost two-thirds of the sex relationships of MSM’s trading sex for money, which may potentially provide a bridge of HIV infection among the MSM general population [24].

The purpose of this study was to examine sexual network mixing patterns that involve HIV-unknown status and risky sexual behavior conditioned on assortative/dissortative mixing by race/ethnicity. Our analysis was based on sexual partnership data for a most at-risk group of MSM, drug-using male sex workers. The study conducted systematic association analysis among an individual’s (ego’s) HIV status, partner’s (alter’s) HIV status based on ego’s knowledge, and the sexual behavior between ego and alter for each combination of sexual partnership formed within and between racial/ethnic groups.

## Methods

### Recruitment and Study Design

Data for this study were collected between May 2003 and February 2004 from drug-using men who traded sex for money with men in Houston, Texas. Participants were recruited using a combination of targeted sampling and participant referral [25–27], described in previously elsewhere [28–30]. Briefly, a purposeful sampling plan was developed wherein key informants who were knowledgeable about male sex work in the city were interviewed to identify neighborhoods and venues with high rates of drug use and/or solicitation of sex-for-money. Once neighborhoods were identified, informants and study personnel were asked to recruit men apparently engaged in sex work as focal participants. Eligibility criteria of a focal participant were: self-identified male at least 17 years old, had exchanged sex for money with another man in the last seven days, and had smoked crack cocaine or injected an illicit substance in the 48 h before being screened. Focal participants were asked to name social, drug-use, and sexual contacts.

From the list of named contacts, contacts were apportioned into strata that consisted of sex/drug associates, friends, paying sexual partners, and other social contacts. Subsequent sampling was weighted toward sex and drug-use contacts. Eligibility criteria for network members referred by the focal or secondary members were: 17 years old and linked to the focal or secondary (referring) participants. The focal participant was given the names of the selected individuals and asked to recruit them as secondary contacts as a means to sample tertiary contacts. Study procedures and data collection instruments were approved by the Committee for the Protection of Human Subjects at the University of Texas Health Science Center at Houston.

### Data

Self-reported data on the socio-demographic characteristics, self-reported histories of HIV/STIs, drug use, and risky sexual behaviors were collected. A sample of 334 males (84.3 %) and 62 females (15.6 %) were interviewed. In addition to information about themselves, these 396 respondents were asked about their contacts’ demographic characteristics, HIV status, risky sexual behaviors, and relationship with the contact. These data produced 4,880 respondent-contact data dyads, with an average of 12 contacts named per respondent (SD = 10, minimum = 1, maximum = 59).

### Analytic Sample

The analytic sample for this study was derived from 257 respondents and consisted of 3,072 male-to-male sexual dyads that had no missing race/ethnic information. Respondents were 33 years old on average (SD = 8, minimum = 18, maximum = 73). Of the respondents, 47 % were White, 45 % Black, and 8 % Hispanic. Almost all, 95 %, had ever traded sex for money, and 91 % had used crack cocaine or cocaine at least once during their lifetimes. Slightly more than half, 52 %, had injected drugs at least once. A quarter, 25 %, reported being HIV-positive. Analytic dyads included 257 respondents who had an average of 12 sex partners (SD = 11; minimum = 1, maximum = 55).

### Measures

Respondents’ (egos’) HIV status was measured by self-report. Participants were asked, “What were the results of your last HIV test?” Results were coded as HIV-positive ego, HIV-negative ego, and, if the respondent’s HIV status was unknown (including indeterminate), as ego unknown. Contacts’ (alters’) HIV status was measured by asking respondents whether they knew the HIV status of their sexual contacts: “Do you think your partner is HIV positive?” Responses were coded HIV-positive alter, HIV-negative alter, and, if unknown, HIV-status unknown.

Dyadic-level risky sexual behavior was measured through respondents’ self-reports of condom use the last time they had sex, “The last time you had sex with [partner], did you (or your partner) use a male condom?” No condom use was coded as unprotected sex (risky sexual behavior). Condom use was coded as protected sex (non-risky sexual behavior). Count data were structured in cross-classified tables by respondents’ HIV status (indexed rows with three levels) and the partners’ HIV status (indexed with three levels), and then stratified by unprotected/protected sexual behavior.

### Assortativity Coefficient

Newman’s discrete assortativity coefficient (*r*) was computed to quantify the level of assortative sexual mixing in the network by discrete characteristics. The mathematical definition is [31]:

$$ r = \frac{Tre - \left\| e^{2} \right\|}{1 - \left\| e^{2} \right\|} $$

where *e* is the matrix whose elements are the cell values $e_{ij}$ and $\left\| {e^{2} } \right\|$ is the sum of the squared values of the elements in the mixing matrix.

The value of *r* lies in a range $-1 \le r \le 1$ with *r* = 1 indicating perfect assortativity. It must be noted that a perfectly dissortative network, *r* = 0, can also approximate a randomly or neutrally mixed network [31]. Assortative mixing coefficients were computed using a pair of three types of HIV statuses for ego and alters (positive, negative, unknown) for marginal and stratified tables. A mixing coefficient of <0.35 was viewed as assortative, between 0.26 and 0.34 as moderately assortative, and between 0.15 and 0.25 as minimally assortative. A coefficient of ≤0.15 was deemed dissortative [32, 33].

### Log-Linear Analysis

Log-linear analysis has been used to model nonrandom selective mixing patterns using contact matrices [34] by estimating different homogeneity/symmetric patterns [35]. Log-linear analysis allows simultaneous analysis of association and interaction patterns among a set of covariates without identifying any variables as a response [36]. A set of log-linear models was estimated to examine the associations among ego’s HIV status (denoted as “E”), alter’s HIV status (denoted as “A”), and unprotected sex (denoted as “U”). It was assumed that this three-way cross-classification has several potential types of independence. Expected cell frequencies for each category of the three variables were modeled (see Table 1) using eight log-linear models [36]. Symbols representing the higher-order term for each variable are shown in square brackets [36]. The models examined were: (1) mutual independence ([E][A][U]), (2) three types of joint independence ([E][AU], [A][EU], [U][EA]), (3) three types of conditional independence ([EA][EU], [EA][AU], [EU][AU]), and [4] homogeneous association ([EA][EU][AU]). Model expressions, corresponding to null hypotheses, interpretations of models, and mathematical formula are described in the Technical Appendix.

**Table 1:** Contingency tables by ego’s and alter’s HIV statuses stratified by risky sexual behavior (protected and unprotected sex) and Newman’s discrete assortativity coefficient

		Protected sex				Unprotected sex				Both protected and unprotected		
		Alter’s HIV				Alter’s HIV				Alter’s HIV		
		Negative	Positive	Unknown		Negative	Positive	Unknown		Negative	Positive	Unknown
(White–White)												
Ego’s HIV	Negative	51	12	370	Negative	59	8	380	Negative	110	20	750
	Positive	7	5	30	Positive	14	15	143	Positive	21	20	173
	Unknown	11	1	74	Unknown	9	4	140	Unknown	20	5	214
Assortativity coefficient		*r*_−+_	*r*_−?_	*r*_+?_		*r*_−+_	*r*_−?_	*r*_+?_		*r*_−+_	*r*_−?_	*r*_+?_
		0.194	−0.003	0.166		0.424	0.041	0.064		0.367	0.020	0.085
(Black–Black)												
Ego’s HIV	Negative	30	2	51	Negative	59	6	116	Negative	89	8	167
	Positive	9	7	26	Positive	23	31	149	Positive	32	38	175
	Unknown	10	0	9	Unknown	26	3	53	Unknown	36	3	62
Assortativity coefficient		*r*_−+_	*r*_−?_	*r*_+?_		*r*_−+_	*r*_−?_	*r*_+?_		*r*_−+_	*r*_−?_	*r*_+?_
		0.421	−0.085	0.103		0.495	0.006	0.063		0.484	−0.014	0.069
(White–Black) or (Black–White)												
Ego’s HIV	Negative	21	3	130	Negative	19	3	170	Negative	40	6	300
	Positive	3	9	38	Positive	4	14	94	Positive	7	23	132
	Unknown	4	0	6	Unknown	4	1	28	Unknown	8	1	34
Assortativity coefficient		*r*_−+_	*r*_−?_	*r*_+?_		*r*_−+_	*r*_−?_	*r*_+?_		*r*_−+_	*r*_−?_	*r*_+?_
		0.625	−0.038	0.051		0.645	−0.008	0.044		0.640	−0.018	0.049
(White–Hispanic) or (Hispanic–White)												
Ego’s HIV	Negative	11	2	94	Negative	15	5	136	Negative	26	7	230
	Positive	2	2	11	Positive	1	14	39	Positive	3	16	50
	Unknown	1	0	5	Unknown	7	6	66	Unknown	8	6	71
Assortativity coefficient		*r*_−+_	*r*_−?_	*r*_+?_		*r*_−+_	*r*_−?_	*r*_+?_		*r*_−+_	*r*_−?_	*r*_+?_
		0.346	−0.008	0.092		0.661	0.002	0.197		0.603	0.000	0.173

*r*_−+_ assortativity coefficient between HIV negative and positive, *r*_−?_ assortativity coefficient between HIV negative and unknown, *r*_+?_ assortativity coefficient between HIV positive and unknown

The fit of the models to the data was evaluated using two statistical procedures. First, parsimony was examined by assessing the best fit among the log-linear models. Best fit was identified by computing deviance statistics (*G* ^2^), corresponding *p* values (*p*), the likelihood-ratio statistic between deviances for candidate models, and the Akaike Information Criterion (*AIC*). Second, estimated parameters of the best-fitted model were estimated. Our log-linear analysis was conducted under the assumption that samples of respondents and contacts were random. Analysis was restricted to the dyads of White–White, Black–Black, White–Black and Black–White, and White–Hispanic and Hispanic–White to minimize the number of sparse cells or cells with no cases.

## Results and Discussion

Table 2 shows the distributions of different combinations of dyads by race/ethnicity and the frequency and percentage of each type of dyads. Approximately half of the dyads were White egos and White alters (43 %), followed by Black–Black (20 %), and Black–White (11 %).

**Table 2:** Frequency and percentage by dyad types

Dyads by race (ego-alter)	Frequency	Percentage	Frequency of ego
**White–White**	**1,333**	**43.39**	**118**
**Black–Black**	**610**	**19.86**	**99**
Hispanic–Hispanic	49	1.60	11
**White–Black**	**221**	**7.19**	**44**
**Black–White**	**330**	**10.74**	**60**
**White–Hispanic**	**191**	**6.22**	**52**
**Hispanic–White**	**226**	**7.36**	**18**
Black–Hispanic	104	3.39	33
Hispanic–Black	8	0.26	4
Total	3,072	100.0	439^a^

^a^Includes overlapped individuals across different combinations of dyads (*N* = 257); bolded rows indicate dyads used for subsequent log-linear analysis

### Assortativity Coefficients

Assortativity by HIV status (positive, negative, unknown) and risky sexual behavior (protected, unprotected sex) is shown in Table 1. Results show assortativity by HIV status (*r*_−+_, negative or positive), regardless of the sexual behavior among Black–Black dyads and White–Black and Black–White dyads. Assortativity by HIV status (*r*_−+_) was strongest for unprotected sex for most race/ethnicity dyad combinations. Among White–Hispanic and Hispanic–White dyads, there was a minimum assortativity by HIV positive and unknown statuses (*r*_+?_) only for unprotected sex.

### Log-Linear Analysis

Table 3 illustrates the results of goodness-of-fit statistics of degree of freedom (*df*), deviance (*G* ^2^) and corresponding *p* value, and *AIC*. The results indicate that the null model of conditional independence ([EA] [EU] between alter’s HIV status, A, and unprotected sex, U, given ego’s HIV status, E, failed to be rejected. This implies that the association between ego’s and alter’s HIV status [EA] does not depend on unprotected sex, or that the association between ego’s HIV status and unprotected sex [EU] does not depend on alter’s HIV status.

**Table 3:** Goodness-of-fit tests for different log-linear models (*df*, deviance statistics (*G* ^2^), *p*-value, and *AIC*)

Dyad types	(White–White)				(Black–Black)			
Model	*df*	*G* ^2^	*p*	*AIC*	*df*	*G* ^2^	*p*	*AIC*
(1) [E][A][U]	12	103.29	<0.001	11.43	12	81.35	<0.001	9.74
(2) [E][AU]	10	102.33	<0.001	11.60	10	74.61	<0.001	9.59
[A][EU]	10	32.84	<0.001	7.73	10	65.49	<0.001	9.08
[U][EA]	8	78.65	<0.001	10.50	8	23.14	<0.01	6.95
**(3) [EA][EU]**	**6**	**8.20**	**=0.224**	**6.81**	**6**	**7.27**	**=0.296**	**6.29**
[EA][AU]	6	77.69	<0.001	10.67	6	16.39	<0.05	6.80
[EU][AU]	8	31.88	<0.001	7.90	8	58.74	<0.001	8.93
**(4) [EA][EU][AU]**	**4**	**7.07**	**=0.132**	**6.97**	**4**	**3.57**	**=0.467**	**2.22**

Dyad types	(White–Black) or (Black–White)				(White–Hispanic) or (Hispanic–White)			
Model	*df*	*G* ^2^	*p*	*AIC*	*df*	*G* ^2^	*p*	*AIC*
(1) [E][A][U]	12	59.53	<0.001	8.05	12	77.64	<0.001	8.79
(2) [E][AU]	10	55.70	<0.001	8.06	10	72.21	<0.001	8.71
[A][EU]	10	45.72	<0.001	7.51	10	34.41	<0.001	6.61
[U][EA]	8	20.35	<0.01	6.32	8	48.58	<0.001	7.62
**(3) [EA][EU]**	**6**	**6.55**	**=0.364**	**5.77**	**6**	**5.34**	**=0.501**	**5.44**
[EA][AU]	6	16.61	<0.05	6.33	9	43.14	<0.001	7.54
[EU][AU]	8	41.98	<0.001	7.52	8	28.98	<0.001	6.53
**(4) [EA][EU][AU]**	**4**	**2.41**	**=0.661**	**5.77**	**4**	**2.75**	**=0.600**	**5.51**

(1) Mutual independence model, (2) joint independence model, (3) conditional independence model, and (4) Homogeneous association model. Goodness of fit results indicate that both conditional independence model for [EA][EU] and homogeneous association model [EA][EU][AU] fit the data well for all dyad types (bolded)

Similarly, the homogeneous association model [EA] [EU] [AU] failed to be rejected. Failure to reject this null hypothesis implies that this model also fits the data well for all dyad types by race/ethnicity. To determine which model, the conditional independence or homogenous association model, provided a better fit to the data, the likelihood ratio test was conducted as the conditional independence model [EA] [EU] was nested within the homogeneous association model. The model was examined to determine whether an additional interaction term [AU] improved the fit of the model. The results indicated that the interaction term [AU] was not statistically significant for all types of dyads, which suggests that the conditional independence model ([EA] [EU]) most accurately described the data. Further, *AIC* values for [EA] [EU] were smaller than or almost identical to those for [EA] [EU] [AU], except for Black–Black dyads, which also supports the choice of this model. Therefore, the conditional independence model ([EA] [EU]) and its corresponding higher order association terms, [EA] and [EU], were further examined.

Table 4 shows the estimated relative risk (*RR*) of the interaction terms based on the best-fit conditional association model [EA] [EU]. The interaction term [EA] represents the association between ego’s HIV status, E, and alter’s HIV status, A, which essentially tests for assortative mixing by HIV status in the dyad. The reference category of HIV status for egos and alters was “HIV-negative.” The reference for sexual behavior was “protected sex.” The results showed a significant association between ego’s and alter’s HIV status, controlling for unprotected sex. Among White–White dyads, the relative risk of having HIV-positive alters increased by 5.23 for HIV-positive egos (*RR* = 5.23, *SE* = 2.07, *p* < 0.001). Similar trends were observed for Black–Black dyads (*RR* = 13.21, *SE* = 5.82, *p* < 0.001), White–Black or Black–White dyads (*RR* = 21.90, *SE* = 13.47, *p* < 0.001), and White–Hispanic or Hispanic–White dyads (*RR* = 19.81, *SE* = 15.05, *p* < 0.001).

**Table 4:** Results of estimated relative risk for the conditional independence log-linear model [EA] [EU]

Term	Interaction	(W–W)	(B–B)	(W–B) or (B–W)	(W–H) or (H–W)
[EA]	(Ego+) × (Alt+)	5.23*** (2.07)	13.21*** (5.82)	21.90*** (13.47)	19.81*** (15.05)
	(Ego+) × (Alt?)	1.21 (0.31)	2.91*** (0.68)	2.51* (1.06)	1.88 (1.19)
	(Ego?) × (Alt+)	1.38 (0.76)	0.93 (0.65)	0.83 (0.96)	2.78 (1.92)
	(Ego?) × (Alt?)	1.57† (0.40)	0.92 (0.23)	0.57 (0.24)	1.00 (0.43)
[EU]	(Ego+) × (U)	3.97*** (0.73)	2.22*** (0.48)	1.80** (0.36)	2.47** (0.78)
	(Ego?) × (U)	1.72*** (0.26)	1.98* (0.57)	2.65* (1.00)	9.03*** (3.98)

Values in parenthesis represent standard errors. “Ego+” and “Alt+” indicate HIV positive status, and “Ego?” and “Alt?” indicate unknown HIV status. “U” indicates unprotected sex (not using condom). Reference category for E (Ego’s HIV status) and A (Alter’s HIV status) is “negative”; reference category for U is protected sex (use condom)

^†^ *p* < 0.1; * p < 0.05; ** *p* < 0.01; *** *p* < 0.001 for two-tailed test

Ego’s HIV positive status was significantly associated with alter’s HIV-unknown status only in dyads that involved Blacks. Among Black–Black dyads, the relative risk of having an HIV-unknown alter increased by 2.91 for HIV-positive egos (*RR* = 2.91, *SE* = 0.68, *p* < 0.001). A similar trend was observed among White-Black or Black-White dyads, although these relative risks were less significant (*RR* = 2.51, *SE* = 1.06, *p* < 0.05). The relative risk of having HIV-positive or HIV-unknown alters relative to HIV-negative alters was not statistically significant for HIV-unknown egos.

Ego’s HIV status was found to be significantly associated with risky sexual behavior [EU], controlling for alter’s HIV status for all dyad types. The relative risk of engaging in unprotected sex was 3.97 (*SE* = 0.73, *p* < 0.001) for HIV-positive egos compared to HIV-negative egos among White–White dyads, 2.22 (*SE* = 0.48, *p* < 0.001), among Black–Black dyads, 1.80 (*SE* = 0.36, *p* < 0.01), among White–Black or Black–White dyads, and 2.47 (*SE* = 0.78, *p* < 0.01), and among White-Hispanic or Hispanic–White dyads. Similarly, the relative risk of engaging in unprotected sex was 1.72 (*SE* = 0.26, *p* < 0.001) for HIV-unknown egos compared to HIV-negative egos among White–White dyads, 1.98 (*SE* = 0.57, *p* < 0.05) for Black–Black dyads, 2.65 (*SE* = 1.00, *p* < 0.05) for White-Black or Black-White dyads, and 9.03 (*SE* = 3.98, *p* < 0.001) for White–Hispanic or Hispanic–White dyads.

## Conclusions

This study examined network mixing by race/ethnicity, HIV status, and sexual behaviors in a sample of most at-risk MSM who use drugs and trade sex for money. The findings of the study suggest that having unprotected sex varied by the racial/ethnic composition of the dyad. In general, assortative mixing by HIV status (positive or negative) was greater when a dyad had unprotected sex than when a dyad had protected sex for all dyad combinations. Black–Black and Black–White (or White–Black) dyads were more likely to display assortative mixing by HIV status (positive or negative), irrespective of their engaging in unprotected sex than other dyad combinations investigated. Moreover, an interaction between race/ethnicity and HIV status was found. The results suggest that ego’s HIV status, rather than alter’s, was associated with unprotected sex. These finding suggest that some network mixing patterns are race/ethnicity specific.

The study found that HIV-positive most at-risk MSM generally tend to engage in risky sexual behavior, especially in an intra-racial sexual partnership. HIV-positive most at-risk Black MSM tend to have unprotected sex and have HIV status unknown partners, especially with Black partners. Under this condition, dissortative mixing, engaging in unprotected sex with a partner of opposite HIV status, is more likely to occur between Black partners. The willingness of HIV-positive most at-risk Black MSM to engage in transactional sex while unaware of their partner’s HIV status and their behavioral patterns may facilitating bridging from the sexually active core of the a network to the less active fringe, thereby contributing to the disproportionate infection rates among Black MSM. HIV-unknown most at-risk MSM compared to those who were HIV-negative also were more likely to have unprotected sex regardless of their partner’s status, especially among Whites. However, findings did not suggest how HIV-unknown most at-risk MSM having sexual relationships with HIV-positive or unknown partners compared with their having sexual relationships with HIV-negative partners.

Our study has several limitations. First, the study used a sample that was, although purposeful, essentially the result of snowball sampling to analyze most at-risk MSM a majority of who used drugs and traded sex for money. Therefore, the results are not generalizable to MSM or to Black MSM in particular. Second, sex was defined as both oral and anal sex. The risk of HIV infection is known to differ greatly between the two behaviors and, thereby, the results should be interpreted with caution. Third, the analysis depended on respondents’ knowledge of their partners’ HIV status, and the validity of the respondents’ knowledge is unknown. Notably, imperfect knowledge may lead to an underestimation of discordant HIV status in the dyads. Fourth, the study did not address network factors that may increase or reduce HIV risk, including seroadaptive practices, such as serosorting or strategic positioning (insertive anal intercourse with discordant partners). No strong evidence of racial/ethnic differences has been reported in seroadaptive behaviors in enhancing disparities in HIV prevalence [37]. Fifth, the modeling approach was limited because factors known to increase HIV transmission in sexual networks, such as dissortative mixing by age [38, 39], were not considered. Although our preliminary analysis found no evidence of dissortative mixing by age, further analysis should include more complex interactions when modeling the dyads.

Despite these limitations, this study provides a different view of sexual mixing patterns by race/ethnicity and HIV in networks of most at-risk MSM. Appropriate intervention efforts should be directed at reducing the disproportionate number of HIV infections among most at-risk Black MSM, especially those who engage in transactional sex and use drugs. Our study proposes intervention programs that increasingly emphasize the importance of HIV testing, inform the available linkage to care and antiretroviral treatment of HIV-positive for most at-risk Black MSM, and encourage serodiscussion, especially among most at-risk Black MSM. Black most at-risk MSM unaware of partners’ status expose potentially HIV-negative partners to the risk of HIV infection via unprotected sexual intercourse. This type of program, however, may not be effective for relationships among most at-risk Whites. Intervention programs for most at-risk White MSM should increasingly emphasize the importance of continuing condom use, especially for those who do not know their own HIV status.

## Technical Appendix

See Table 5.

**Table 5:** Log-linear independence models for three-dimensional tables, corresponding null hypotheses, and formulae

(1) Mutual independence model: [E][A][U]—No relationship exists among E, A, and U $log\mu_{ijk} = \lambda + \lambda_{i}^{E} + \lambda_{j}^{A} + \lambda_{k}^{U}$ where $\mu_{ijk}$ is expected cell counts, $\lambda_{i}^{E}$ is a main effect of E, $\lambda_{j}^{A}$ is a main effect of A, $\lambda_{k}^{U}$ is a main effect of U
(2) Joint independence models: [E][AU]*—*A and U are jointly independent of E $log\mu_{ijk} = \lambda + \lambda_{i}^{E} + \lambda_{j}^{A} + \lambda_{k}^{U} + \lambda_{jk}^{AU}$ where $\lambda_{jk}^{AU}$ is an association term that represents interaction between A and U [A][EU]—E and U are jointly independent of A $log\mu_{ijk} = \lambda + \lambda_{i}^{E} + \lambda_{j}^{A} + \lambda_{k}^{U} + \lambda_{ik}^{EU}$ where $\lambda_{ik}^{EU}$ is an association term that represents interaction between E and U [U][EA]—E and A are jointly independent of U $log\mu_{ijk} = \lambda + \lambda_{i}^{E} + \lambda_{j}^{A} + \lambda_{k}^{U} + \lambda_{ij}^{EA}$ where $\lambda_{ij}^{EA}$ is an association term that represents interaction between E and A
(3) Conditional independence model: [EA][EU]—A and U are conditionally independent, given E. $log\mu_{ijk} = \lambda + \lambda_{i}^{E} + \lambda_{j}^{A} + \lambda_{k}^{U} + \lambda_{ij}^{EA} + \lambda_{ik}^{EU}$ where $\lambda_{ij}^{EA}$ is an association term that represents interaction between E and A, $\lambda_{ik}^{EU}$ represents interaction between E and U [EA][AU]—E and U are conditionally independent, given A $log\mu_{ijk} = \lambda + \lambda_{i}^{E} + \lambda_{j}^{A} + \lambda_{k}^{U} + \lambda_{ij}^{EA} + \lambda_{jk}^{AU}$ where $\lambda_{ij}^{EA }$ is an association term that represents interaction between E and A, $\lambda_{jk}^{AU}$ represents interaction between A and U [EU][AU]—E and A are conditionally independent, given U $log\mu_{ijk} = \lambda + \lambda_{i}^{E} + \lambda_{j}^{A} + \lambda_{k}^{U} + \lambda_{ik}^{EU} + \lambda_{jk}^{AU}$ where $\lambda_{ik}^{EU }$ is an association term that represents interaction between E and U, $\lambda_{jk}^{AU}$ represents interaction between A and U
(4) Homogeneous association model: [EA][EU][AU]—Conditional association between any pairs of variables is identical given the third one. $log\mu_{ijk} = \lambda + \lambda_{i}^{E} + \lambda_{j}^{A} + \lambda_{k}^{U} + \lambda_{ij}^{EA} + \lambda_{ik}^{EU} + \lambda_{jk}^{AU}$ where $\lambda_{ij}^{EA}$ is an association term that represents interaction between E and A, $\lambda_{ik}^{EU}$ is an association term that represents interaction between E and U, $\lambda_{jk}^{AU}$ is an association term that represents interaction between A and U

In the case of model fit: (1) Mutual independence implies that ego’s HIV-status (E), alter’s HIV-status (A), and unprotected sex (U) are independent of one another (marginal independence). (2) Joint independence [E] [AU], as an example, implies that neither alter’s HIV-status (A) nor unprotected sex status (U) has an effect on ego’s HIV-status (E). (3) Conditional independence [EA] [EU], as an example, implies that any relationships that exist between alter’s HIV-status (A) and unprotected sex status (U) can be explained by ego’s HIV-status (E). (4) Homogeneous association model [EA] [EU][AU] implies that conditional association between any pairs of variables is identical given the third one. For more detailed information, refer to Agresti (2013) [36]
